# Determinants of stillbirth among deliveries conducted at west Shoa zone public hospitals, central Ethiopia: a case–control study

**DOI:** 10.1186/s12887-024-04953-2

**Published:** 2024-07-27

**Authors:** Fayisa Abdisa Tufa, Delelegn Yilma, Dereje Yadesa, Meseret Robi Tura

**Affiliations:** 1 Department of Public Health, College of Health Sciences and Referral Hospital, Ambo University, Ambo, Ethiopia; 2 Department of Medicine, College of Health Sciences and Referral Hospital, Ambo University, Ambo, Ethiopia; 3 Department of Nursing, College of Health Sciences and Referral Hospital, Ambo University, P.O. Box 19, Ambo, Ethiopia

**Keywords:** Determinants, Stillbirth, Case‒Control, Ethiopia

## Abstract

**Background:**

Globally, 2.6 million stillbirths are estimated to occur each year. The causes of stillbirth are often unknown but can be attributed to various causes. Therefore, identifying the determinants of stillbirth is quite important for applying further meaningful interventions. The purpose of this study was to identify the determinants of stillbirth among deliveries conducted at selected public hospitals in the West Shoa Zone, Oromia, Ethiopia.

**Methods:**

A hospital-based unmatched case‒control study with a 1:4 ratio was conducted. A total of 431 (87 cases and 344 controls) participants were involved. A systematic random sampling method was used for control selection. Data were collected using interview administered questionnaire and analysed using SPSS version 26 software. Binary logistic regression analyses were performed for the independent variables and outcome variables. Adjusted odds ratios (AORs) with 95% confidence intervals (CIs) were estimated to assess the strength of the associations, and statistical significance was declared at *P* value < 0.05.

**Results:**

In this study, 428 mothers who delivered (85 patients and 343 controls) participated, for a 99.3% response rate. Preeclampsia/eclampsia (AOR = 13.43, 95% CI: 5.67–31.82), other health conditions (AOR = 5.39, 95% CI: 2.34–12.46), mal-presentation (AOR = 3.42, 95% CI: 1.50–7.76), umbilical cord accidents (AOR = 2.57, 95% CI: 1.11–5.93), meconium-stained amniotic fluid problems (AOR = 5.01, 95% CI: 2.15–11.67) and low birth weight (AOR = 2.91, 95% CI: 1.28–6.59) were identified as determinant variables of stillbirth.

**Conclusions:**

Low birth weight, referral status, meconium-stained amniotic fluid problems, umbilical cord accidents, mal-presentation and preeclampsia/eclampsia were identified as independent determinants of stillbirth. Therefore, hospitals and health workers are recommended to focus on identifying and preventing these factors.

## Introduction

A stillbirth occurs when a fetus passes away in the uterus either before or during delivery. It is a baby that is born beyond 1000 g in weight or after 28 weeks of gestation without showing any signs of life. A stillbirth is the birth of a baby after the 24th week of pregnancy that, after being taken from the mother entirely, did not breathe or exhibit any other indication of life [1–3].

When comparing stillbirths among countries, the World Health Organizations (WHO) advises reporting cases involving birthweights of 1000 g or more, gestational ages of 28 weeks or more, or body lengths of 35 cm or more. On the basis of their newborn care service standards, these nations do not, however, consistently follow the same set of guidelines for reporting stillborn children. [1, 4–6].

An estimated 2.6 million stillbirths take place annually throughout the world. Ninety-eight percent of cases take place in low- and middle-income nations. Over 40% of stillbirths happen during labor. Better, more considerate delivery care, such regular check-ups and prompt access to emergency obstetric care when necessary, could have prevented this tragedy [7–9].

In 2014, the World Health Assembly approved all of the Newborn Action Plans (ENAPs), which included a global goal of 12 stillbirths per 1000 live births worldwide by 2030. 128 nations, the majority of which were high- and upper-middle-income, had achieved this goal by 2019, however many of them had not [1, 8]. This plan may appear to be an impossible goal to achieve given that the documented reduction during the Millennium Development Goal (MDG) period was only from 24.7 to 18.4 per 1000 births. In 2019, there were 14 stillbirths per 1000 total births, implying that one stillbirth occurred every 16 s. Furthermore, 75% of stillbirths occur in sub-Saharan Africa and Southeast Asia [1, 7, 10].

Stillbirth rates range from 20 to 40 per 1000 births in sub-Saharan and Southeast Asian countries, which is ten times higher than in developed regions. The incidence of stillbirth resulting from intrapartum death is higher in poor nations (59% versus 10%) compared to developed nations. According to earlier data, Finland had 2 stillbirths for every 1000 live births, whereas Pakistan and Ethiopia had 40 and 25.5 per 1000, respectively, indicating the differences in maternal care standards. Even though stillbirths make up a sizable fraction of perinatal deaths, there is not enough emphasis on addressing this issue, as shown by the 2015 Sustainable Development Goals (SDG) [4, 11–13]. Between 1990 and 2016, Ethiopia cut its maternal death rate in half, and between 1990 and 2015, it cut its neonatal mortality rate from 55 per 1000 live births to 28 per 1000 live births. Advances in technology, a rise in skilled healthcare providers, and the growth of healthcare facilities all contributed to this achievement [14–16].

Furthermore, many studies have shown that stillbirth in general and intrapartum stillborn neonates are caused by maternal sociodemographic factors, medical and obstetric conditions, access to quality obstetric care services during pregnancy, pregnancy type, timing, and quality of intrapartum care, and fetal factors [1, 17, 18]. Stillbirth is a sorrowful occurrence for not only the newborn, but also the mother and father, the extended family, the health care system, and the community as a whole. Stillbirth during pregnancy or childbirth is a tragic event that is not effectively addressed in global agendas, regulations, or sponsored programs. Women and their families face psychological expenses, including maternal depression, financial implications and ramifications, stigma and taboos [10].

However, the significance of the loss and its impact on the grieving mother or parents is sometimes overlooked. There is growing evidence that many fatalities are potentially preventable, and stillbirth rates are becoming regarded as an important indicator of care quality. Overall, stillbirth is a sensitive indicator of both the healthcare system and the quality of care provided throughout pregnancy and childbirth. Stillbirth has a varied influence. It creates psychological depression in women and affects the daily lives of family members and health-care providers. The causes of stillbirth are frequently unknown, but can be related to a variety of causes. Different medical conditions, including hypertension, metabolic disorders, infections and nutritional deficiencies, can emerge or become aggravated during pregnancy as critical factors that could cause adverse pregnancy outcomes, including stillbirth [4, 18]. Ethiopia reduced maternal mortality by half between 1990 and 2016, and neonatal mortality decreased from 55 per 1000 to 28 per 1000 live births between 1990 and 2015. This progress was made possible by the expansion of health-care facilities, an increase in competent providers, and technological advancements [14–16].

Understanding the causes of stillbirth will be crucial for gathering timely representative data and carrying out every new-born action plan aimed at reducing preventable stillbirths to less than 12 per 1000 live births (15, 16, and 19). The number of studies undertaken in the country on stillbirth factors is limited; they also concentrated on a single environment, making it unable to examine the topic thoroughly. Studies on this topic are uncommon in the Oromia region, especially in the study area.

## Method and materials

### Study area and study period

The study was conducted at a public hospital in the West Shoa Zone. West Shewa zone is one of the zones found in the Oromia regional state. Ambo is the zonal town where the Zonal Health Office was founded. The town lies approximately 114 km from the capital of Addis Ababa. The estimated current total population of the Zone is 2,661,188, of whom 49% are men and 51% are women. Oromo is the largest ethnic group, accounting for 93.82%. Afaan Oromo is the largest spoken language in this zone. This Zone has 22 districts and 572 kebeles/gandas, and the zone has nine public hospitals, 91 health centers, 1 private higher clinic, 40 medium private clinics, 168 small clinics, 43 drug stores, 21 drug vendors and 4 pharmacies. The study was carried out from December 13, 2022–March 13, 2023.

### Study design

A facility-based unmatched case‒control study was also conducted.

### Population

The source population were all mothers who gave birth in public hospitals in the west Shoa zone and the study population were mothers who gave birth after 28 weeks of pregnancy at the selected public hospitals during the data collection period. Cases were all mothers who gave stillbirth at the selected public hospitals during the period from December 13, 2022 to March 13, 2023. The control group comprises mothers with live births during the study period at the selected public hospitals. Mothers who were critically ill were excluded.

### Sample size determination

The sample size was calculated by the two-population proportion formula using Epi-Info version 7 for unmatched case‒control studies. To calculate sample size, the main exposure variables, such as mode of admission, duration of labor, preceding birth interval and meconium-stained amniotic fluid, were considered based on previous studies on the determinants of stillborn neonates. The assumption used to calculate sample size was as follows: two-sided confidence interval (CI) = 95%, power = 80%, and ratio of cases to controls = 1:4. Among these variables, meconium-stained amniotic fluid was chosen as the alternative determinant variable of stillbirth, with a percentage of controls exposed of 10.8% and an odds ratio of 2.67 according to an unmatched case‒control study performed in the Oromia and Bale zones [14]. According to this assumption, the calculated sample size was 392**,** and by adding a 10% nonresponse rate, the final sample size was 431 (87 cases and 344 controls) (Table 1).

**Table 1:** Sample Size Calculated by Using Epi-Info Version 7 for Unmatched Case–Control Study Conducted on Determinants of Stillbirth among Delivered Mothers in Selected Public Hospitals Located in West Shoa Zone, Oromia, Ethiopia, 2023

S/N	Variables	Confidence interval	Power	Control-case ratio	Prop-of Cases	Prop- of Controls	Odd ratio	Sample Size
1	Meconium stained amniotic fluid	95%	80%	4:1	36.6	10.8	2.67	392
2	Duration of labor	95%	80%	4:1	41.8	23.9	3.21	170
3	Mode Of admission	95%	80%	4:1	71.6	39.6	3.17	180
4	Preceding birth interval	95%	80%	4:1	54.1	33.0	2.99	188

### Sampling technique

**Fig. 1:**
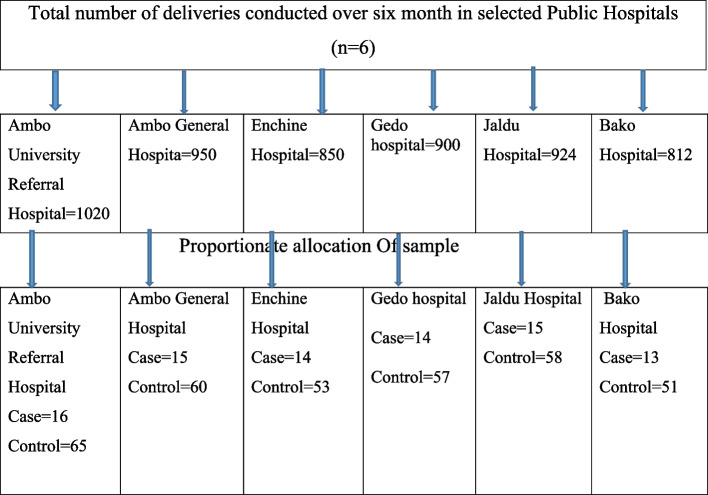
**S**chematic presentation that shows sampling technique for deliveries conducted in selected public hospital of West Shewa zone, Oromia, Ethiopia, 2023

In the West Shoa Zone, there are nine hospitals, six of which were selected by simple random sampling methods. These hospitals included Ambo University Referral Hospital, Ambo General Hospital, Jaldu Hospital, Bako Hospital, Gedo Hospital, and Enchine Hospital. The sample size was allocated to the study hospitals proportionally based on the average delivery report of six months prior to data collection. Among the 87 stillborn mothers who delivered at the Ambo University Referral Hospital over six months, 1020 were stillborn. In the Ambo General Hospital, 950 of the 83 deliveries conducted over six months were still births. At Jaldu Hospital, 924 deliveries were conducted over six months, of which 82 were still births. In Gedo Hospital, 900 total deliveries were conducted over six months, of which 78 were still births. In Enchine Hospital, 850 out of the 76 deliveries conducted over six months were still births, and in Bako Hospital, 812 total deliveries conducted over six months out of the 72 deliveries were still births. In total, 5456 deliveries were conducted over six months in these six hospitals. The number of stillborn patients was 478. The sample was proportionally allocated for all selected public hospitals based on data from the previous six-month total reports of deliveries. Since a number of patients were manageable, all patients were included in the study until the required number of patients was met. However, a systematic random sample technique was used to select controls among the eligible study participants (Fig. 1).

### Dependent variable

Stillbirth outcomes.

### Independent variables

#### The sociodemographic factors

included maternal age, maternal residence, marital status, educational status and occupational status.

#### Maternal health and pregnancy-related factors

Parity, Preceding Birth Interval, Anaemia, Diabetes, Antenatal Follow Visit, Nutritional Dietary Counselling, RH Incompatibility Antepartum Haemorrhage, Preeclampsia/Eclampsia, Gestational Age, Pregnancy Intention, Previous Adverse Birth Outcomes and Multiple Pregnancies.

#### Labour and delivery factors

Mode of Admission, Premature Rupture of Membrane, Use of Partograph, Foetal Malpresentation, Mode of Labour, Obstructed Labour, Labour Duration, Mode of Delivery, and Umbilical Cord Accident During Delivery.

#### Fetal-related factors, namely

Fetal birth weight, presence of the mealconium-stained amniotic fluid problem and presence of congenital anomalies, were found to be independent variables.

### Operational definition

#### Cases

Delivers whose birth outcome was stillborn were defined as babies born without any signs of life at or after 28 weeks of gestation [21].

#### Controls

deliveries whose birth outcome was live births, which were defined as babies showing evidence of life (such as beating of the heart or pulsation of the umbilical cord) at delivery or after 28 weeks of gestation [21].

#### Macerated stillbirth

In the context of this study, macerated stillbirths were defined as the death of a foetus before delivery with skin degeneration [3].

#### Fresh stillbirths

In the context of this study, a foetus who died during labour was considered a fresh stillborn foetus [3].

### Data collection tools and procedures

After reviewing the different related literature, the questionnaire was developed as appropriate to address the study objectives [14, 18, 22, 25]. The questionnaire has four parts: sociodemographic factors, maternal health and pregnancy-related factors, labor and delivery factors and fetal-related factors. The data were collected in the labor ward via face-to-face interviews via structured questionnaires from all eligible mothers and cross-checking card records of mothers by 6 midwives who were working in selected public hospitals, and one senior midwife was assigned as a supervisor.

### Data quality control and management

The questionnaire was translated from English to Afaan Oromo and then retranslated to English to check its consistency by language experts. Both the data collectors and a supervisor were trained for one day on the objective of the study and techniques of data collection prior to actual data collection. Pretests with 5% of the total sample size were conducted at Guder Hospital, which is located in the West Shoa Zone and is not included in the study sample, to administer the questionnaire. All questionnaires and card records completed by the data collectors were examined for completeness, consistency and corrected errors by a supervisor to ensure the quality of the data. During the data collection time, regular monitoring and supervision of the overall activity were performed by the supervisor and principal investigator to ensure the quality of the data. All the collected data were checked, cleaned and coded to avoid inconsistencies and incompleteness before analysis.

### Data processing and analysis

The collected data were checked for completeness, coded, cleaned, explored and exported to SPSS Version 26 for analysis. The data were described in terms of frequency, mean and percentage and then presented in terms of tables, text and graph descriptions; then, logistic regression was performed. The statistical significance and strength of the associations between independent variables and an outcome variable were measured by a binary logistic regression model. Variables with *P* values less than 0.25 in the bivariable logistic regressions were transferred to the multivariable logistic regression model to adjust for confounding effects. The Hosmer and Lemeshow goodness of fit was used to test model fitness. A multi-collinearity test was performed using variance inflation factor (VIF) to assess the correlation between independent variables and no multi-collinearity was detected. Adjusted odds ratios with 95% confidence intervals (CIs) were estimated to assess the strength of the associations, and statistically significant differences were defined as those for which the 95% CI and *P* value were < 0.05 for multivariate analysis.

## Results

### Socio-demographic characteristics of the study participants

In this study, a total of 431 (87 cases and 344 controls) study participants with a case-to-control ratio of 1:4 were enrolled. A total of 428 (85 cases and 343 controls) respondents participated in the study, for a 99.3% response rate. The mean age of the study participants was 27.20 years (SD ± 5.93 years for cases) and 26.40 years (SD ± 5.59 years for controls). Approximately half of the women (223, 52.1%) were aged between 20 and 35 years. Among these patients, 37 (43.5%) were cases, and 186 (54.2%) were controls. Most of the study participants, 50 (58.8%) of the patients and 238 (68.6%) of the controls, lived in rural areas. Regarding the educational status of the mothers, 32 (36.4%) of the cases and 140 (40.8%) of the controls had completed secondary school. Nearly three-fourths of the 70 (82.3%) patients and 275 (80.1%) of the controls were housewives. Regarding the marital status of the participants, 77 (90.5%) of the mothers and 329 (95.9%) of the controls were married (Table 2).

**Table 2:** Socio-Demographic Characteristics Of The Mothers Delivered In Selected Public Hospitals Located In West Shoa Zone, Oromia, Ethiopia, 2023

Variables	Categories	Stillbirth Outcome		
		Yes = Cases(*n* = 85)	No = Controls(*n* = 343)	Total
		Frequency(%)	Frequency(%)	Frequency%)
Age	< 20 Years	25(29.4)	93(27.1)	118(27.6)
	20–35	37(43.5)	186(54.2)	223(52.1)
	> 35	23(27.0)	64(18.6)	87(20.3)
Residence	Urban	35(41.2)	104(30.3)	139(32.7)
	Rural	50(58.8)	238(68.6)	288(67.3)
Level Of Educational	No Formal Education	5(5.7)	20(5.8)	25(5.8)
	Primary School	32(36.4)	137(40.0)	169(39.5)
	Secondary School	32(36.4)	140(40.8)	172(40.2)
	Collage And Above	16(18.8)	50(14.6)	66(15.4)
Occupational Status	House Wife	70(82.3)	275(80.1)	345(80.6)
	Employer	5(5.8)	14(4.8)	19(4.4)
	Private Business	6(7.0)	37(10.7)	43(12.5)
	Student	4(4.7)	17(4.9)	21(4.9)
Marital Status	Single	2(2.3)	3(0.9)	5(1.2)
	Married	77(90.5)	329(95.9)	406(94.8)
	Widowed	3(3.5)	4(1.7)	7(1.6)
	Divorced	3(3.5)	7(2.0)	10(2.3)

### Maternal health and pregnancy-related factors

Most of the study participants, 82 (96.5%) of the cases and 335 (97.6%) of the controls’ pregnancy intentions, were planned and supported. Most of the respondents, 52 (61.2%) of the patients and 267 (77.8%) of the controls, had more than four ANC follow-up visits. The majority of participants, 65 (76.5%) of the patients and 303 (88.1%) of the controls, had a gestational age greater than or equal to 37 weeks. Regarding the number of pregnancies, 14 (16.5%) of the patients and 25 (7.3%) of the controls had multiple pregnancies. Approximately 63 (74.1%) of the patients had preeclampsia/eclampsia, whereas 63 (18.4%) of the controls had preeclampsia. Approximately 43 (50.6%) of the cases and 95 (27.7%) of the control study respondents had a preceding birth interval of < 24 months. Concerning the parity status of the mothers, the majority, 61 (71.8%) of the cases and 227 (66.2%) of the controls, were multiparous. Among the study participants, 73 (85.8%) of the patients and 310 (90.4%) of the controls received nutritional counselling during pregnancy. Approximately 15 (17.6%) of the case respondents and 26 (7.6%) of the control respondents experienced antepartum hemorrhage (APH) during their current pregnancy. Most of the study participants, 79 (92.9%) of the patients and 320 (93.3%) of the controls, had no previous adverse birth outcomes. Similarly, approximately 80 (94.2%) of the patients and 332 (96.8%) of the controls had no RH incompatibility (Table 3).

**Table 3:** Maternal Health And Pregnancy Related Factors Of Study Participants Among Deliveries Conducted At Selected Public Hospitals In West Whoa Zone, Oromia, Ethiopia, 2023

Variables	Categories	Stillbirth outcome		
		Yes = Cases(*n* = 85)	Controls = (*n* = 343)	Total
		Frequency(%)	Frequency (%)	Frequency(%)
Pregnancy Intention	planned and supported	82(96.5)	335(97.6)	417(97.4)
	Unplanned $ unsupported	3(3.5)	8(2.3)	11(2.5)
ANC Follow Up visit	= > 4 visit	33(38.8)	76(22.2)	109(25.5)
	< 4 visit	52(61.2)	267(77.8)	319(74.5)
Gestational Age of birth	< 37 weeks	20(23.5)	41(11.9)	61(14.2)
	= > 37 weeks	65(76.5)	303(88.1)	368(86.0)
Multiple pregnancy	Yes	14(16.5)	25(7.3)	39(9.1)
	No	71(83.5)	318(92.7)	389(90.8)
Preeclampsia/Eclampsia	Yes	63(74.1)	63(18.4)	126(29.4)
	No	22(25.8)	280(81.6)	302(70.6)
Parity Status	Primipara	3(3.5)	34(9.9)	37(8.6)
	Mult-para	61(71.8)	227(66.2)	288(67.3)
	Grandmulti-	4(4.7)	44(12.8)	48(11.2)
Previous Adverse Birth Outcome	Yes	6(7.0)	23(6.7)	29(6.7)
	No	79(92.9)	320(93.3)	399(93.2)
Preceding Birth Interval	< 24 months	43(50.6)	95(27.7)	138(32.2)
	= > 24 months	42(49.4)	246(71.7)	288(67.3)
Diabetes	Yes	2(2.3)	6(1.7)	8(2.0)
	No	83(92.7)	327(95.3)	410(95.8)
Anemia	Yes	5(5.8)	10(2.9)	15(3.5)
	No	80(94.2)	333(97.0)	413(96.5)
Rh Incompatibility	Yes	5(5.8%)	11(3.2)	16(3.7)
	No	80(94.2)	332(96.8)	412(96.3)
Nutritional Counseling During Pregnancy	Yes	73(85.8)	310(90.4)	383(89.5)
	No	12(14.1)	33(9.6)	45(10.5)
APH During Current Pregnancy	Yes	15(17.6)	26(7.6)	41(9.3)
	No	70(82.3)	317(92.4)	387(90.4)
Premature Membrane Rupture	Yes	4(4.7)	12(3.4)	16(3.7)
	No	81(95.3)	331(96.5)	412(96.3)

### Labor and delivery-related factors

Among the study participants, 62 (72.9%) of the patients and 92 (26.8%) of the controls were referred from other health facilities. Eighty (94.2%) of the cases and 328 (95.6%) of the controls had spontaneous labor. During this study, 18 (21.2%) of the patients and 42 (12.2%) of the controls had a duration of labor greater than 18 h. During delivery, in 79 (91.8%) of the patients and in 318 (92.7% of the controls) mothers, the three components of the partograph (fetal condition, progress of labor, and maternal condition) were completed. The majority of participants, 76 (89.4%) of the patients and 310 (90.4%) of the controls, delivered via spontaneous vaginal delivery/SVD/. Fetal mal-presentation occurred in 50 (58.8%) of the patients and 68 (19.8%) of the controls. In more than half of the study participants, 46 (54.1%) had umbilical cord accidents during delivery, while 78 (22.7%) had cord accidents (Table 4).

**Table 4:** Labor And Delivery Related Factors Of Study Participants Among Deliveries Conducted At Selected Public Hospitals Located In West Whoa Zone, Oromia, Ethiopia, 2023

Variables	Categories	Stillbirth outcome		
		Yes = Cases(*n* = 85)	No = Controls(*n* = 343)	Total
		Frequency(%)	Frequency(%)	Frequency(%)
Mode of admission	Referral	62(72.9)	92(26.8)	154(36.0)
	Non referral	23(27.0)	251(73.2)	274(64.0)
Mode of labor	Spontaneous	80(94.2)	328(95.6)	408(95.3)
	Induced	5(5.8)	15(4.3)	20(4.7)
Duration of labor	> 18 h	18(21.2)	42(12.2)	60(14.0)
	= < 18 h	67(78.8)	301(87.7)	368(86.0)
Obstructed labor	Yes	7(8.2)	12(3.5)	19(4.4)
	No	78(91.8)	331(96.5)	409(95.6)
Parthograph use	Yes	79(92.9)	318(92.7)	3971(92.7)
	No	6(7.0)	25(7.3)	31(7.2)
Mode of deliver	SVD	76(89.4)	310(90.4)	386(90.2)
	Forceps assisted	4(4.7)	7(2.0)	11(2.6)
	CS	5(5.8)	26(7.6)	31(7.2)
Fetal mal-presentation	Yes	50(58.8)	68(19.8)	118(27.6)
	No	35(41.2)	275(80.2)	310(72.4)
Cord accident	Yes	46(54.1)	78(22.7)	124(29.0)
	No	39(45.9)	265(77.2)	304(71.0)

### Fetal-related characteristics

Among the study participants, 15 (17.6%) had cases, and 25 (7.3%) had cases. of the controls had congenital malformations. The majority (55, 64.7%) of the patients had meconium-stained amniotic fluid problems, whereas 55 (16.0%) of the controls had meconium-stained amniotic fluid problems. Approximately 53 (62.3%) of the cases and 90 (26.2%) of the controls had birth weights less than 2500 g (Table 5).

**Table 5:** Labor And Delivery Related Factors Of Study Participants Among Deliveries Conducted At Selected Public Hospitals Located In West Whoa Zone, Oromia, Ethiopia, 2023

Variables	Categories	Stillbirth outcome		
		Yes = Cases(*n* = 88)	No = Controls(*n* = 354)	Total
		Frequency(%)	Frequency(%)	Total n(%)
Congenital anomalies	Yes	15(17.6)	25(7.3)	40(9.3)
	No	70(82.3)	318(92.7)	388(90.6)
Meconium stained amniotic fluid problem	Yes	55(64.7)	55(16.0)	110(25.7)
	No	30(35.3)	288(84.0)	318(74.3)
Birth Weight of babies	< 2500gm	53(62.3)	90(26.2)	149(33.4)
	= > 2500gm	32(37.6)	253(73.7)	285(65.6)

### Determinants of stillbirth

The associations of stillbirth with different independent variables were assessed via bivariate logistic regression and multivariate logistic regression analyses. The associations of stillbirth with different independent variables were assessed via bivariate logistic regression to identify possible determinants of stillbirth, and variables with *p* values < 0.25 were included in the multivariable logistic analysis. Accordingly, fifteen [15] variables were selected based on the selection criteria. Based on the results of the bivariate logistic regression analysis, the sociodemographic characteristics of the mothers, gestational age, preceding birth interval, number of ANC follow-up visits, multiple pregnancy, APH during the current pregnancy, preeclampsia/eclampsia were from the maternal health and pregnancy-related factors, mode of admission/referral status, duration of labor, obstructed labor, fetal malpresentation, umbilical cord accidents during delivery were from labor- and delivery-related factors, and maternal amniotic fluid problems, congenital anomalies and baby birth weights were filtered out from fetal-related factors and showed a statistical association with stillbirth in the bivariate logistic regression analysis.

After those variables with *P* < 0.25 were entered into the multivariate logistic regression analysis, the results showed that having a malpresentation, preeclampsia/eclampsia, being referred from another health facility, having an umbilical cord accident during delivery, having a baby’s birth weight less than 2500 g and having a meconium-stained amniotic fluid problem were significantly associated with stillbirth at the 95% confidence intervals, with *p* values < 0.05.

The odds of preeclampsia/eclampsia were 13 times higher among cases (stillbirths) compared to controls (live births); (AOR = 13.43, CI = 5.67–31.82). The odds of mothers referred from other health facilities were 5.39 (AOR = 5.39, 95% CI = 2.34–12.46) times higher among cases (stillbirths) compared to controls (live births). The odds of mothers who experienced umbilical cord accidents during delivery were 2.57 (AOR = 2.57, 95% CI = 1.11–5.93) times higher among cases (stillbirths) compared to controls (live births). The odds of mothers whose baby birth weights less than 2500gm were 2.91 (AOR = 2.92, 95% CI = 1.28–6.59) times higher among cases (stillbirths) compared to controls (live births). In addition, the odds of mothers who had a malpresentation (AOR = 3.42, 95% CI = 1.50–7.76) were three times higher among cases (stillbirths) compared to controls (live births). The odds of mothers who had meconium-stained amniotic fluid volume problems (AOR = 5.01, 95% CI = 2.15–11.67) were also 5 times higher among cases (stillbirths) compared to controls (live births) (Table 6).

**Table 6:** Bivariable And Multivariable Logistic Regression Analysis of Factors Associated With Stillbirth, West Shoa Zone, Oromia, Ethiopia, 2023

Variables	Categories	Stillbirth Outcome				
		Yes = Cases N(%)	No = Controls N(%)	COR(95%CI)	AOR(95%CI)	*P*-Value
		Frequency(%)	Frequency(%)			
Residence	Rural	50(58.8)	238(68.6)	0.63(0.39–1.02)	0.74(0.31–1.76)	0.49
	Urban	35(41.2)	104(30.3)	1		
Gestational age	< 37 week	20(23.5)	41(11.9)	2.10(1.17–3.75)	1.50(0.53–4.27)	0.44
	= > 37 week	65(76.5)	303(88.1)	1	1	
Preceding birth interval	< 24 month	43(50.6)	95(27.7)	4.03(2.37–6.83)	2.26(0.97–5.27)	0.06
	= > 24 month	42(49.4)	246(72.3)	1		
ANC follow up visit	< 4 visit	33(38.8)	76(22.2)	1.99(1.21–3.27)	1.90(0.80–4.50)	0.15
	= > 4 visit	52(61.2)	267(77.8)	1	1	
Multiple pregnancy	Yes	14(16.5)	25(7.3)	2.11(1.06–4.20)	2.19(0.54–8.88)	0.27
	No	71(83.5)	318(92.7)	1	1	
Eclampsia	Yes	63(74.1)	63(18.4)	11.89(6.90–20.48)	13.43(5.67–31.82)*	0.00
	No	22(25.8)	280(81.6)	1	1	
Duration of labor	> 18 h	18(21.2)	42(12.2)	1.64(0.90–2.98)	1.73(0.62–4.82)	0.29
	= < 18 h	67(78.8)	301(87.7)	1	1	
APH during current pregnancy	Yes	15(17.6)	26(7.6)	2.59(1.31–5.14)	2.03(0.67–6.19)	0.21
	No	70(82.3)	317(92.4)	1	1	
Mode of admission	Referral	63(72.9)	92(26.8)	6.32(3.78–10.56)	5.39(2.34–12.46)*	0.00
	Nonreferral	22(27.1)	251(73.2)	1	1	
Obstructed labor	Yes	7(8.2)	12(3.5)	2.46(0.94–6.45)	2.28(0.29–17.86)	0.43
	No	78(91.8)	331(96.5)	1	1	
Fetal malpresentation	Yes	50(58.8)	68(19.8)	5.31(3.23–8.71)	3.42(1.50–7.76)*	0.00
	No	35(41.2)	275(80.2)	1	1	
Cord accident during delivery	Yes	46(54.1)	78(22.7)	3.74(2.30–6.10)	2.57(1.11–5.93)*	0.03
	No	39(45.9)	265(77.2)	1	1	
Congenital anomalies	Yes	15(17.6)	25(7.3)	2.70(1.45–5.37)	2.40(0.68–8.49)	0.18
	No	70(82.3)	318(92.7)	1	1	
Meconium stained Amniotic fluid	Yes	55(64.7)	55(16.0)	8.57(5.12–14.36)	5.01(2.15–11.67)*	0.00
	No	30(35.3)	288(84.0)	1	1	
Babies weight	< 2500 g	53(62.3)	95(26.2)	4.33(2.65–7.06)	2.91(1.28–6.59)*	0.01
	= > 2500 g	32(37.6)	253(73.7)	1	1	

1 = Indicate Reference Category, * = statistically significant at *p* value < 0.05

## Discussion

Stillbirth has a wide range of consequences for family members and health-care providers. Identifying the causes of stillbirth is critical for mitigating this problem and assisting couples in having a healthy baby. As a result, this unmatched case–control study identified the determinants of stillbirth among deliveries conducted at public hospitals in the west Shoa zone, Oromia, central Ethiopia, by incorporating the most immediate factors influencing birth outcomes in the study setting. Accordingly, this study revealed that having preeclampsia/eclampsia during pregnancy, being referred from other health facilities, having an umbilical cord accident during delivery, having fetal malpresentation during labor, having a baby’s birth weight less than 2500 g, and having meconium-stained amniotic fluid problems were identified as determinants of stillbirth.

In this study, mothers with maternal preeclampsia/eclampsia had a greater risk of stillbirth than their male counterparts. Those mothers with maternal hypertension were 13.43 times more likely to have stillborn mothers than those who did not have hypertension during pregnancy. This finding is consistent with previous research in southern Ethiopia, northern Ethiopia, the central Tigray and Oromia region, and the northern Shoa zone [21–23]. This could be due to poorly controlled hypertension during pregnancy, which can lead to placental abruption, utero placental insufficiency, placental infarction, or fetal-maternal hemorrhage. Maternal hypertension may reduce fetal growth for these reasons. Fetal-maternal hemorrhage increases maternal serum levels offetoprotein, which has been linked to stillbirth.

Compared to their counterparts in this study, mothers who were referred from other health facilities had five times greater odds of stillbirths than those who were not referred. This finding is consistent with the findings of Cameron and in the bale zone of Ethiopia [14, 24]. This could be because the majority of referred patients come from rural peripheral health facilities and may have serious complications. The distance to the hospitals to which they were referred contributes to delays in receiving care, which can obviously cost their fetus's life. This finding contrasts with a study conducted at Jima University Specialized Hospital [25], which revealed a reduction in stillbirth among pregnant women referred from another health facility compared to those who were not referred. The disparity could be attributed to differences in study area, and/or sample size.

The odds of mothers who experienced malpresentation during labor were three times greater for those who experienced stillbirth than for those who did not. This result agreed with the findings of a study conducted in northern Uganda and in our country’s Oromia region, the northern Shoa zone [6, 22]. Such findings could be the result of obstetric complications such as prolonged labor, obstructed labor, premature membrane rupture, and cord prolapse.

The odds of having a stillbirth were five times greater for mothers with meconium-stained amniotic fluid than for their counterparts in the current study. This finding is consistent with findings in sub-Saharan Africa and in Ethiopia and in the central Tigray and Oromia bale zones [3, 14, 21]. This could be due to the intrauterine passage of meconium into amniotic fluid. However, meconium aspiration syndrome causes respiratory failure and ultimately results in fetal loss [3].

The results of the present study revealed that stillbirths were nearly three times more likely to occur when compared with those of newborns weighing less than 2500gm. These results are consistent with research from Cameron and our country, Tigray Central Zone, Oromia, in the Bale Zone [14, 21, 24]. This could be because low birth weight is caused by maternal complications such as hypertension or diabetes mellitus that occur before or during pregnancy. In addition, long-term maternal malnutrition, poor health, and inadequate prenatal care could result. Low birth weight is a complex public health issue that can lead to premature birth death, which is called stillbirth.

The odds of stillbirth were two times greater among mothers who experienced umbilical cord accidents during delivery than among those who did not experience cord accidents during delivery. This finding is consistent with findings in sub-Saharan Africa [26]. This could be due to cord compression, knots, torsions/strictures, cord prolapse, vasa previa, and compromised fetal microcirculation, which cause fetal distress and lead to fetal death.

### Strength and limitations of the study

The study used a case–control study design, which is appropriate for addressing the research question and enables the identification of possible determinants of stillbirth. Both cases and controls were recruited from the same health facility to avoid context of variation among participants. Since it is an institution-based study, its generalizability is not strong enough (mothers who did not come to health institutions were excluded). There might be recall bias because mothers who had stillbirth are more likely to remember their exposure than mothers who have live births.

## Conclusion and recommendations

In the present study, preeclampsia, referral from other health facilities, meconium-stained amniotic fluid problems, umbilical cord accidents during delivery, fetal malpresentation and fetal low birth weight were identified as determining factors of stillbirth. The identified determinants are manageable and can be amenable to interventions. Therefore, close monitoring and appropriate prevention strategies during antepartum, intrapartum and early referral systems should be implemented to address these risk factors for stillbirth. Likewise, identifying pregnant women who have danger signs of maternal complications, such as labor complications, should be prioritized.

We recommended to health professionals that mothers with hypertension before and during pregnancy be encouraged to seek health care early without delay. It is recommended that mothers who are at high risk of maternal complications be given priority. Health workers at labor and delivery should provide routine monitoring of mothers and fetal conditions during labor. We also recommended for the hospitals to early referral systems for mothers with danger signs of maternal complications are recommended and emergency obstetric care facilities and important diagnostic materials should be provided during labor care and delivery. Future researchers better to conduct studies supplemented with qualitative data.

## Data Availability

The datasets used and/or analysed during the current study available from the corresponding author on reasonable request.

## Abbreviations

ANC: Ante Natal Care
AOR: Adjusted Odds Ratio
COR: Crude Odd Ratio
ENAP: Newborn action plan
LBW: Low Birth Weight
WHO: World Health Organization
